# Albumin-To-Alkaline Phosphatase Ratio as a Novel and Promising Prognostic Biomarker in Patients Undergoing Esophagectomy for Carcinoma: A Propensity Score Matching Study

**DOI:** 10.3389/fonc.2021.764076

**Published:** 2021-10-20

**Authors:** Xianying Zhu, Dongni Chen, Shuangjiang Li, Wenbiao Zhang, Yongjiang Li, Xiaoyu Wang, Jian Zhou, Zhesheng Wen

**Affiliations:** ^1^ State Key Laboratory of Oncology in South China, Collaborative Innovation Center for Cancer Medicine, Sun Yat-Sen University Cancer Center, Guangzhou, China; ^2^ Intensive Care Unit, Sun Yat-Sen University Cancer Center, Sun Yat-Sen University, Guangzhou, China; ^3^ Department of Thoracic Surgery, Nanfang Hospital, Southern Medical University, Guangzhou, China; ^4^ Department of Endoscopy and Laser, Sun Yat-Sen University Cancer Center, Sun Yat-Sen University, Guangzhou, China; ^5^ Department of Medical Imaging, Sun Yat-Sen University Cancer Center, Sun Yat-Sen University, Guangzhou, China; ^6^ Department of Nuclear Medicine, Sun Yat-Sen University Cancer Center, Sun Yat-Sen University, Guangzhou, China; ^7^ Department of Thoracic Oncology, Sun Yat-Sen University Cancer Center, Sun Yat-Sen University, Guangzhou, China

**Keywords:** albumin-to-alkaline phosphatase ratio, esophageal squamous cell carcinoma, prognosis, prediction, esophagectomy

## Abstract

**Background:**

Albumin-to-alkaline phosphatase ratio (AAPR) has been reported as a novel prognostic predictor for numerous solid tumors. We aimed to assess the prognostic role of preoperative AAPR in surgically resectable esophageal squamous cell carcinoma (ESCC) by a propensity score matching (PSM) analysis with predictive nomograms.

**Methods:**

Our study was conducted in a single-center prospective database between June 2009 and December 2012. Kaplan-Meier analysis was used to distinguish the difference in survival outcomes between patients stratified by an AAPR threshold. Multivariable Cox proportional hazards regression model was finally generated to specify independent prognostic markers for the entire and PSM cohorts.

**Results:**

A total of 497 patients with ESCC were included in this study. An AAPR of 0.50 was determined as the optimal cutoff point for prognostic outcome stratification. Patients with AAPR<0.50 had significantly worse overall survival (OS), and progression-free survival (PFS) compared to those with AAPR≥0.50 (Log-rank *P*<0.001). This significant difference remained stable in the PSM analysis. Multivariable analyses based on the entire and PSM cohorts consistently showed that AAPR<0.50 might be one of the most predominant prognostic factors resulting in unfavorable OS and PFS of ESCC patients undergoing esophagectomy (*P*<0.001). The nomograms consisting of AAPR and other independent prognostic factors further demonstrated a plausible predictive accuracy of postoperative OS and PFS.

**Conclusion:**

AAPR can be considered as a simple, convenient and noninvasive biomarker with a significant prognostic effect in surgically resected ESCC.

## 1 Introduction

### 1.1 Background

Esophageal cancer (EC) ranks seventh in global cancer incidence and sixth in cancer-specific mortality (1). EC can be subdivided into esophageal adenocarcinoma and esophageal squamous cell carcinoma (ESCC). The latter is extremely prevalent across the EC hotspots worldwide, accounting for approximately 90% of all histological subtypes (2). Despite recent advances in the development of minimally invasive techniques, optimization of chemoradiotherapy regimens, and innovations in molecularly targeted therapies, the post-treatment prognosis of EC has remained discouraging in decades, with 5-year survival rates ranging from 10% to 20% (3, 4). Esophagectomy, as the mainstay of curative treatment for EC, is still considered a life-threatening gastrointestinal procedure with high mortality rates ranging from 8% to 23% (5–7). Therefore, it becomes crucial to delve into a series of putative prognostic markers that can accurately predict the outcome of the procedure and help to draw up an individualized treatment plan in advance for EC patients intending to undergo esophagectomy.

There is growing evidence that systemic immuno-nutritional status, which can be conveniently and effectively characterized by peripheral blood biomarkers, plays a pivotal role in the pathogenesis and progression of cancers, and further contributes to being a determinant of cancer-related prognosis (4, 8–11). Serum albumin (sALB), a traditional serum biochemical marker synthesized by the liver, has been widely used to reflect the nutritional reserve of the host and considered as a prognostic indicator for malignant tumors (12–14). Alkaline phosphatase (ALP), another hydrolase widely accepted as a disease marker of the hepatobiliary system, bone, and kidney, has also been correlated with postoperative survival of cancer patients (15). In summary, a novel and simple scoring system by integrating sALB with serum ALP, named albumin-to-alkaline phosphatase ratio (AAPR), has been constantly validated as a reliable prognostic indicator across a wide range of malignant tumors, regardless of their treatment options (12, 16–24). However, to our knowledge, the potential prognostic value of AAPR has not yet been explored in patients undergoing esophagectomy for EC.

### 1.2 Objectives

Considering these issues, the purpose of this propensity score matching (PSM) study was to provide a comprehensive assessment of preoperative AAPR as a validated prognostic biomarker for surgically resectable ESCC.

## 2 Materials and Methods

### 2.1 Study Design and Protocol

Our study was a PSM analysis performed in the ESCC database prospectively maintained in our cancer center. We received formal approval from the Institutional Review Board (No: GZR 2018-120) and performed all relevant procedures compliant with the Declaration of Helsinki. Finally, we completed this report following the Strengthening the Reporting of Cohort Studies in Surgery (STROCSS) 2019 Guideline (25).

### 2.2 Participant Selection

#### 2.2.1 Settings

We evaluated clinical and survival data of consecutive patients who underwent esophagectomy for ESCC in our inpatient unit between June 2009 and December 2012.

#### 2.2.2 Inclusion and Exclusion Criteria

The following criteria were performed to determine the suitability of patients for inclusion or exclusion:

(i) Patients with surgically resectable ESCC diagnosed by routine histology were included. Esophageal adenocarcinoma or benign diseases would be excluded;
(ii) Esophagectomy *via* Ivor-Lewis, Sweet and McKeown procedures and esophageal reconstruction with a gastric tube were included. Transhiatal esophagectomy would be excluded;
(iii) Peripheral hematological and biochemical indices must be obtained within seven days prior to the procedure. To ensure the objectivity and accuracy of the analyzed data, the scarcity of complete medical records and follow-up characteristics would not be considered;
(iv) Patients receiving neoadjuvant chemotherapy or chemoradiotherapy would not be considered to minimize potential confounding effects caused by changes in peripheral blood composition after preoperative anticancer therapy, which might complicate the prognostic impact of AAPR;
(v) Patients with concurrent or prior malignancies would not be considered, with the aim to avoid the additional assumption of carcinomatous tissues outside of the esophagus on the host immuno-nutritional reserve, and thus attenuate potential bias risks in the prognostic factor analysis.

### 2.3 Follow-Up Survey

Regular follow-up assessments began on the day of surgery and then during in-hospital observation, every three months for the first two years, every six months for the next three years, and then annually until death or the final follow-up date of December 2018. Follow-up assessments included routine laboratory tests, computed tomography scans of head and neck, chest and abdomen, and endoscopy when necessary.

### 2.4 Measures and Definitions of Outcome Data

#### 2.4.1 Patient Characteristics

(i) Basic patient information included: age (years), sex (male/female), and body mass index (BMI, kg/m^2^);
(ii) Patients’ preoperative comorbidities included: gastrointestinal comorbidities (including chronic gastritis, gastroduodenal ulcers, gastroduodenal bleeding, gastroesophageal reflux disease, caustic stricture of the esophagus, and alimentary tract polyps), cardiovascular comorbidities (including hypertension, coronary artery disease, valvular heart disease, cerebrovascular disease, cardiac arrhythmias, chronic heart failure and peripheral artery disease), diabetes mellitus, respiratory comorbidities (including chronic obstructive pulmonary disease, emphysema, tuberculosis, pneumonia, asthma, bronchiectasis and interstitial lung diseases), hepatobiliary comorbidities (including cirrhosis, hepatitis B, hepatitis C, severe fatty liver and cholelithiasis);
(iii) Pathological parameters of the patients included: grade of differentiation (well/moderate/poor), vascular invasion (present/absent), lymphatic invasion (present/absent), perineural and neural invasion (present/absent), tumor size (cm), tumor invasion (T stage), lymph node metastasis (LNM, N stage) and TNM stage. Tumor staging was estimated by our experienced pathologists according to the 8^th^ edition of the AJCC/UICC TNM staging system of ESCC (26).

#### 2.4.2 Acquisition of Peripheral Blood Markers

Blood sampling for each included patient was performed by our experienced nurses within seven days prior to esophagectomy. The following peripheral blood biomarkers would be collected from complete blood counts and biochemical tests: sALB (g/L), ALP (U/L), C-reactive protein (CRP, mg/L), neutrophil count (10^9^/L), and lymphocyte count (10^9^/L). AAPR was calculated by dividing the level of sALB by the level of serum ALP (sALB/ALP). Also, neutrophil to lymphocyte ratio (NLR) was derived from the total numbers of neutrophils and lymphocytes and used for further survival analysis.

#### 2.4.3 Grouping Criteria of AAPR

We used a free online statistical tool constructed on the R framework (https://molpathoheidelberg.shinyapps.io/CutoffFinder_v1/), the *Cutoff Finder*, to determine the optimal cutoff point for AAPR in predicting postoperative survival. We could then compare clinicopathological characteristics and surgical outcomes of the two groups of patients stratified by this threshold of AAPR. In addition, prognostic significance of AAPR was further analyzed in all subgroups that were stratified according to patients’ clinicopathological characteristics.

#### 2.4.4 Outcomes of Interest

The primary endpoint and the secondary endpoint for survival outcome evaluation were overall survival (OS) and progression-free survival (PFS), respectively. OS was defined as the time interval between esophagectomy and the date of death from any cause or censor at final follow-up. PFS was defined as the time interval between esophagectomy and the date of radiological or histological detection of cancerous recurrence or metastasis (4, 27).

Another one secondary objective was to estimate the differences in postoperative complications, 30-day mortality and 90-day mortality between the two AAPR groups. Postoperative complications were defined as any Clavien-Dindo classification grade ≥ III complication occurred during hospitalization (28). Thirty-day mortality and 90-day mortality were defined as any death within 30 and 90 days after surgery.

### 2.5 Surgical Procedure and Combined Treatment Modality

McKeown, Sweet, and Ivor-Lewis esophagectomies were all standard surgical approaches for treating ESCC at our institution. In addition, reconstruction of the gastric tube with an anastomosis in the cervical incision was generally performed through the posterior mediastinal route. A two-field lymphadenectomy was indicated for middle to lower thoracic ESCC, while a three-field lymphadenectomy was indicated for upper thoracic ESCC. Finally, a feeding tube was routinely placed into the stomach or duodenum at the end of the procedure.

Regarding adjuvant therapy after esophagectomy, a detailed chemotherapy regimen based on cisplatin plus fluoropyrimidine and/or radiotherapy would be finalized according to patients’ baseline conditions and pathological findings.

### 2.6 Statistical Analysis

The following statistical methods in our study were completed using IBM SPSS 26.0 software (IBM SPSS Statistics, Version 22.0. Armonk, NY: IBM Corp) and R Studio version 4.0.3 (R Foundation for Statistical Computing, Vienna, Austria). Statistical significance was indicated by a two-sided test *P* value less than 0.050.

We used Pearson’s chi-square test, Yates’ correction test and Fisher exact test to compare categorical data and Mann-Whitney *U* test to compare continuous data [mean ± standard deviation (SD); median and interquartile range (IQR)], respectively.

Kaplan-Meier curve with Log-rank test was applied to analyze the differences in OS and PFS among different patient groups.

We further performed time-dependent receiver operating characteristic (t-ROC) analysis, a special ROC analysis for time-to-event variables, to estimate the ability of AAPR, sALB, ALP, NLR, and CRP to discriminate between the predictive accuracy of postoperative survival throughout the follow-up period. In addition, their areas under curve (AUCs) would be inferred and compared.

In our PSM analysis, a nearest neighbor matching algorithm by caliper matching with a specified distance at 0.20 SD of the logarithm of the propensity score was utilized to help adequately balance the baseline characteristics between the two AAPR groups. Finally, a 1:1 pair of well-matched patients was generated based on their propensity scores. Furthermore, neither sALB nor ALP, critical factors in the direct establishment of AAPR, would be considered in the PSM procedure (12).

The relationships between all estimated characteristics and survival outcomes were initially explored through univariable Cox proportional hazards regression analysis. Thereafter, any clinicopathological parameter with *P*<0.15 was included in multivariable Cox proportional hazards regression models, and hazard ratios (HRs) with 95% confidence intervals (CIs) were then generated to indicate which factors could play a significant prognostic role in predicting OS and PFS.

We further displayed nomograms graphically mapping multivariable Cox proportional hazards regression models built on independent prognostic contributors. In the prognostic nomogram, each predictive covariable was assigned a score from 0 to 100 based on its point scale bar, and the probability of survival was then represented by the cumulative points mapped by all these covariables. In addition, we computed Harrell’s concordance statistic (C-statistic) to measure the goodness-of-fit of each multivariable Cox proportional hazards regression model. Finally, we plotted calibration curves to reveal the consistency between survival outcomes predicted by nomograms and the real-world survival outcomes.

## 3 Results

### 3.1 Patient Demographics and Outcomes

#### 3.1.1 Participant Population and Primary Demographics

**Figure 1:**
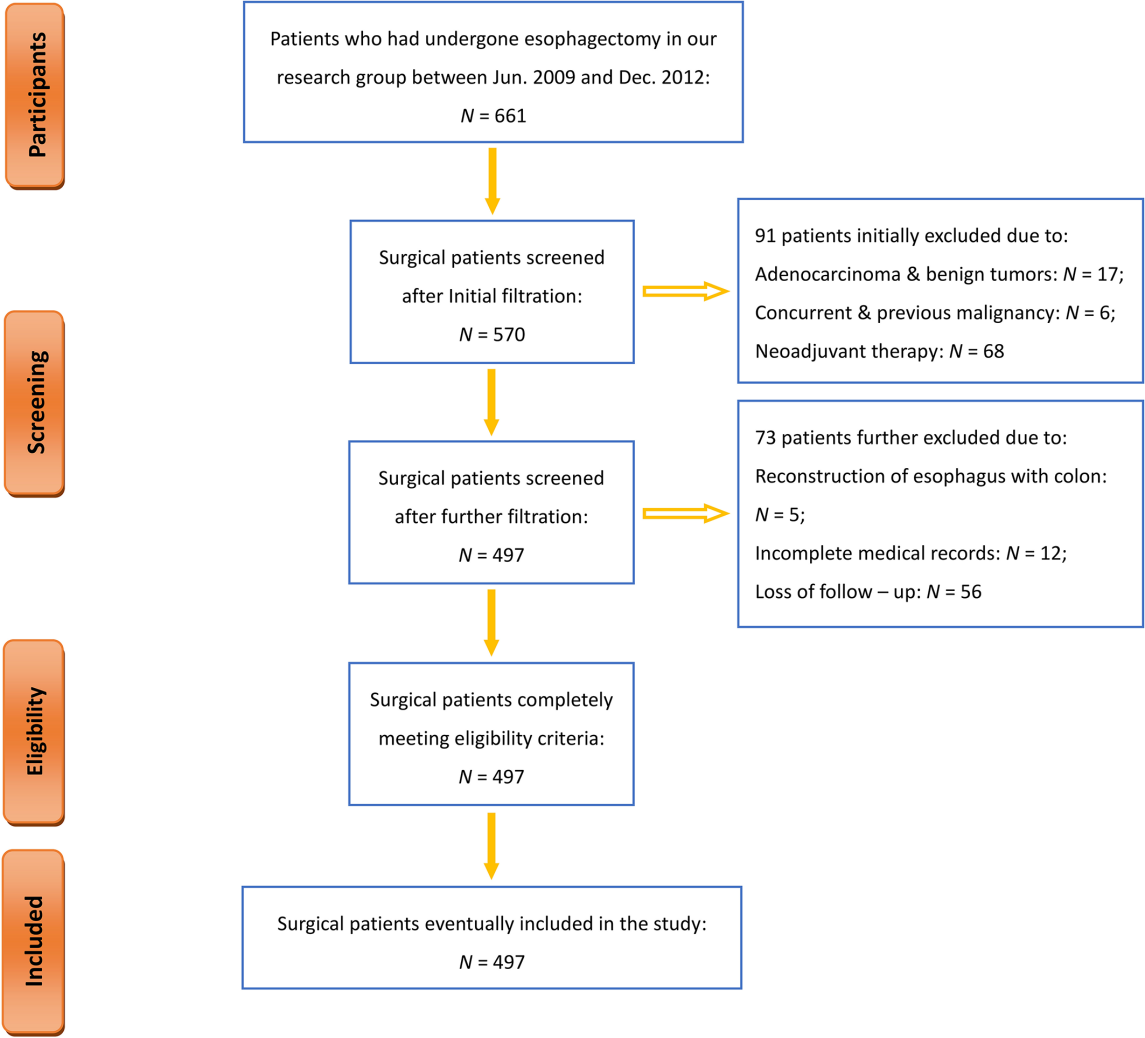
Flowchart of the inclusion and exclusion of participants.

From June 2009 to December 2012, a total of 661 patients underwent esophagectomy at an inpatient unit in our institution. **Figure 1** shows a flowchart of the entire process of participant selection. During the study period, 497 patients have met all eligibility criteria, completed the entire follow-up assessment, and were ultimately enrolled in the current study (**Figure 1**). Their primary demographic data are summarized in **Table 1**.

**Table 1:** Patient characteristics and in-hospital outcomes of the entire cohort.

Characteristics	Entire Cohort			*P* Value
	Total (*N* = 497)	AAPR ≥ 0.50 (*N* = 411)	AAPR < 0.50 (*N* = 86)	
Age (Years)				
Mean ± SD	59.15 ± 9.29	59.11 ± 9.38	59.34 ± 8.92	0.91
Median (IQR)	59 (53 - 66)	59 (53 - 65)	58 (54 - 66)	
Gender (n, %)				
Female	93 (18.7%)	78 (19.0%)	15 (17.4%)	0.74
Male	404 (81.3%)	333 (81.0%)	71 (82.6%)	
Body Mass Index (kg/m^2^)				
Mean ± SD	21.51 ± 3.24	21.69 ± 3.28	20.67 ± 2.89	0.003
Median (IQR)	21.26 (19.47 - 23.56)	21.47 (19.59 - 23.72)	20.20 (18.41 - 22.28)	
Gastrointestinal Comorbidity (n, %)				
Absent	468 (94.2%)	385 (93.7%)	83 (96.5%)	0.31
Present	29 (5.8%)	26 (6.3%)	3 (3.5%)	
Cardio-Cerebrovascular Comorbidity (n, %)				
Absent	427 (85.9%)	353 (85.9%)	74 (86.0%)	0.97
Present	70 (14.1%)	58 (14.1%)	12 (14.0%)	
Diabetes Mellitus (n, %)				
Absent	483 (97.2%)	400 (97.3%)	83 (96.5%)	0.96
Present	14 (2.8%)	11 (2.7%)	3 (3.5%)	
Respiratory Comorbidity (n, %)				
Absent	475 (95.6%)	392 (95.4%)	83 (96.5%)	0.86
Present	22 (4.4%)	19 (4.6%)	3 (3.5%)	
Hepatobiliary Comorbidity (n, %)				
Absent	478 (96.2%)	394 (95.9%)	84 (97.7%)	0.63
Present	19 (3.8%)	17 (4.1%)	2 (2.3%)	
Neutrophil to Lymphocyte Ratio				
Mean ± SD	2.65 ± 1.61	2.58 ± 1.65	2.96 ± 1.40	0.004
Median (IQR)	2.26 (1.70 - 3.17)	2.21 (1.68 - 3.00)	2.65 (1.87 - 3.91)	
C - Reactive Protein (mg/L)				
Mean ± SD	6.16 ± 11.44	5.40 ± 10.18	9.80 ± 15.73	< 0.001
Median (IQR)	2.33 (1.01 - 5.60)	2.05 (0.91 - 4.66)	4.95 (2.32 - 9.56)	
AAPR Value				
Mean ± SD	0.65 ± 0.17	0.69 ± 0.15	0.44 ± 0.06	< 0.001
Median (IQR)	0.62 (0.54 - 0.74)	0.66 (0.59 - 0.76)	0.46 (0.40 - 0.48)	
Differentiation Grade (n, %)				
Well	116 (23.3%)	95 (23.1%)	21 (24.4%)	0.77
Moderate	226 (45.5%)	185 (45.0%)	41 (47.7%)	
Poor	155 (31.2%)	131 (31.9%)	24 (27.9%)	
Vascular Invasion (n, %)				
Absent	457 (92.0%)	380 (92.5%)	77 (89.5%)	0.37
Present	40 (8.0%)	31 (7.5%)	9 (10.5%)	
Lymphatic Invasion (n, %)				
Absent	464 (93.4%)	386 (93.9%)	78 (90.7%)	0.28
Present	33 (6.6%)	25 (6.1%)	8 (9.3%)	
Perineurium & Neural Invasion (n, %)				
Absent	468 (94.2%)	392 (95.4%)	76 (88.4%)	0.012
Present	29 (5.8%)	19 (4.6%)	10 (11.6%)	
Tumor Size (cm)				
Mean ± SD	3.79 ± 1.57	3.67 ± 1.57	4.33 ± 1.45	< 0.001
Median (IQR)	4.0 (3.0 - 5.0)	3.5 (2.5 - 4.5)	4.0 (3.5 - 5.0)	
T Stage (n, %)				
T_1_	54 (10.9%)	49 (11.9%)	5 (5.8%)	0.25
T_2_	92 (18.5%)	75 (18.2%)	17 (19.8%)	
T_3_	351 (70.6%)	287 (69.8%)	64 (74.4%)	
N Stage (n, %)				
N_0_	237 (47.7%)	201 (48.9%)	36 (41.9%)	0.58
N_1_	219 (44.1%)	178 (43.3%)	41 (47.7%)	
N_2_	24 (4.8%)	18 (4.4%)	6 (7.0%)	
N_3_	17 (3.4%)	14 (3.4%)	3 (3.5%)	
TNM Stage (n, %)				
I	95 (19.1%)	76 (18.5%)	19 (22.1%)	0.67
II	248 (49.9%)	205 (49.9%)	43 (50.0%)	
III	154 (31.0%)	130 (31.6%)	24 (27.9%)	
Adjuvant Chemotherapy (n, %)				
Not Received	322 (64.8%)	266 (64.7%)	56 (65.1%)	0.94
Received	175 (35.2%)	145 (35.3%)	30 (34.9%)	
Adjuvant Radiotherapy (n, %)				
Not Received	441 (88.7%)	366 (89.1%)	75 (87.2%)	0.62
Received	56 (11.3%)	45 (10.9%)	11 (12.8%)	
Postoperative Complications (n, %)				
Absent	409 (82.3%)	345 (83.9%)	64 (74.4%)	0.035
Present	88 (17.7%)	66 (16.1%)	22 (25.6%)	
30 - Day Mortality (n, %)				
Absent	494 (99.4%)	408 (99.3%)	86 (100%)	1.0
Present	3 (0.6%)	3 (0.7%)	0 (0%)	
90 - Day Mortality (n, %)				
Absent	484 (97.4%)	403 (98.1%)	81 (94.2%)	0.095
Present	13 (2.6%)	8 (1.9%)	5 (5.8%)	

AAPR, Albumin to Alkaline Phosphatase Ratio; IQR, Interquartile Range; SD, Standard Deviation.

#### 3.1.2 Patient Characteristics

The entire cohort included 404 (81.3%) male and 93 (18.7%) female patients with a mean age of 59.15 ± 9.29 years and a mean BMI of 21.51 ± 3.24 kg/m^2^. A total of 125 (25.2%) patients were diagnosed preoperatively with one or more types of comorbidities (**Table 1**). Postoperative pathological evaluation showed that 95 (19.1%) patients, 248 (49.9%) patients and 154 (31.0%) patients were classified as TNM stage I, II and III ESCC, respectively. There were 52.3% of the cohort (260 patients) confirmed with the presence of LNM (N_1-3_ stage). Other pathological records are also detailed in **Table 1**. Finally, 189 (38.0%) patients received adjuvant chemotherapy (175) and/or adjuvant radiotherapy (56) after esophagectomy.

#### 3.1.3 Laboratory Markers

The mean values regarding sALB, ALP, CRP, neutrophil count, and lymphocyte count in all included patients were 43.04 ± 3.22 g/L, 70.57 ± 19.19 U/L, 6.16 ± 11.44, 4.67 ± 1.87 10^9^/L, and 1.96 ± 0.61 10^9^/L. In addition, both AAPR and NLR could thus be obtained with mean values of 2.65 ± 1.61 and 0.65 ± 0.17, respectively, as shown in **Table 1**.

#### 3.1.4 AAPR Evaluation

**Figure 2:**
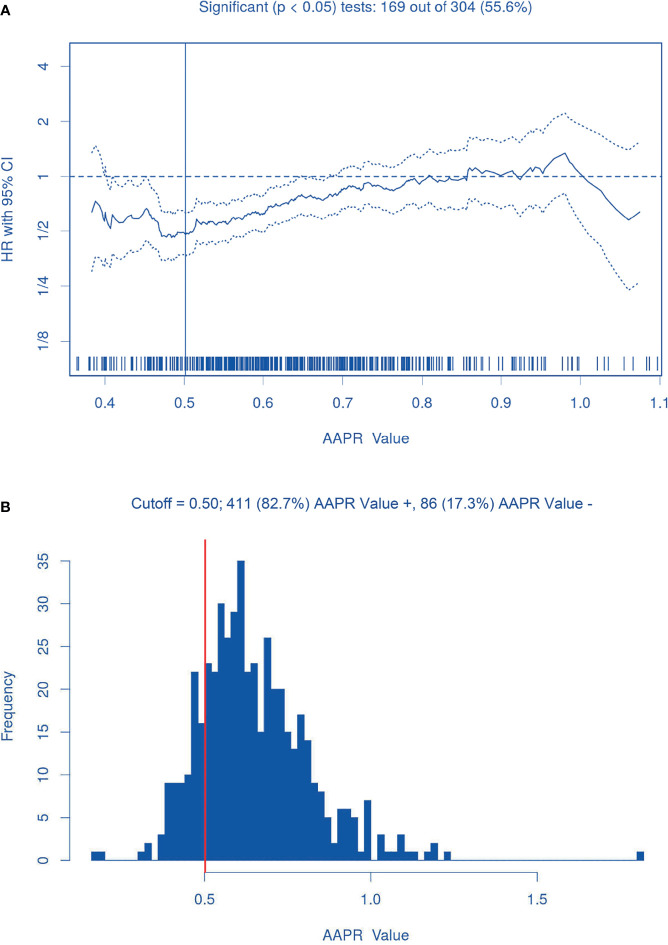
The AAPR optimal cutoff point (0.50). **(A)** Hazard ratios for overall survival according to the cutoff points for preoperative AAPR; **(B)** Patient distribution according to preoperative AAPR. The vertical line indicates the threshold value of AAPR with the most significant split in Log-rank test.

On the basis of biostatistical results generated by the *Cutoff Finder*, various cutoff points for AAPR were statistically significant for prognostic prediction, and an AAPR of 0.50 was finally recognized as the optimal cutoff point, as shown in **Figure 2A**. Therefore, 86 (17.3%) patients were classified into the AAPR<0.50 group, and the remaining 411 (82.7%) patients were classified into the AAPR≥0.50 group (**Table 1** and **Figure 2B**).

#### 3.1.5 Postoperative Outcomes

The median follow-up time for our surgical cohort was 53 (1-114) months. Until the final follow-up date, the rates of OS and PFS were 42.7% and 38.2%, respectively. In-hospital outcomes are also detailed in **Table 1**. There were 88 (17.7%) patients developed with Clavien-Dindo grade ≥ III complications in the perioperative period. The 30-day and 90-day mortality rates for the entire cohort were 0.6% (3 cases) and 2.6% (13 cases), respectively.

### 3.2 Discriminatory Power of Peripheral Blood Biomarkers

**Figure 3:**
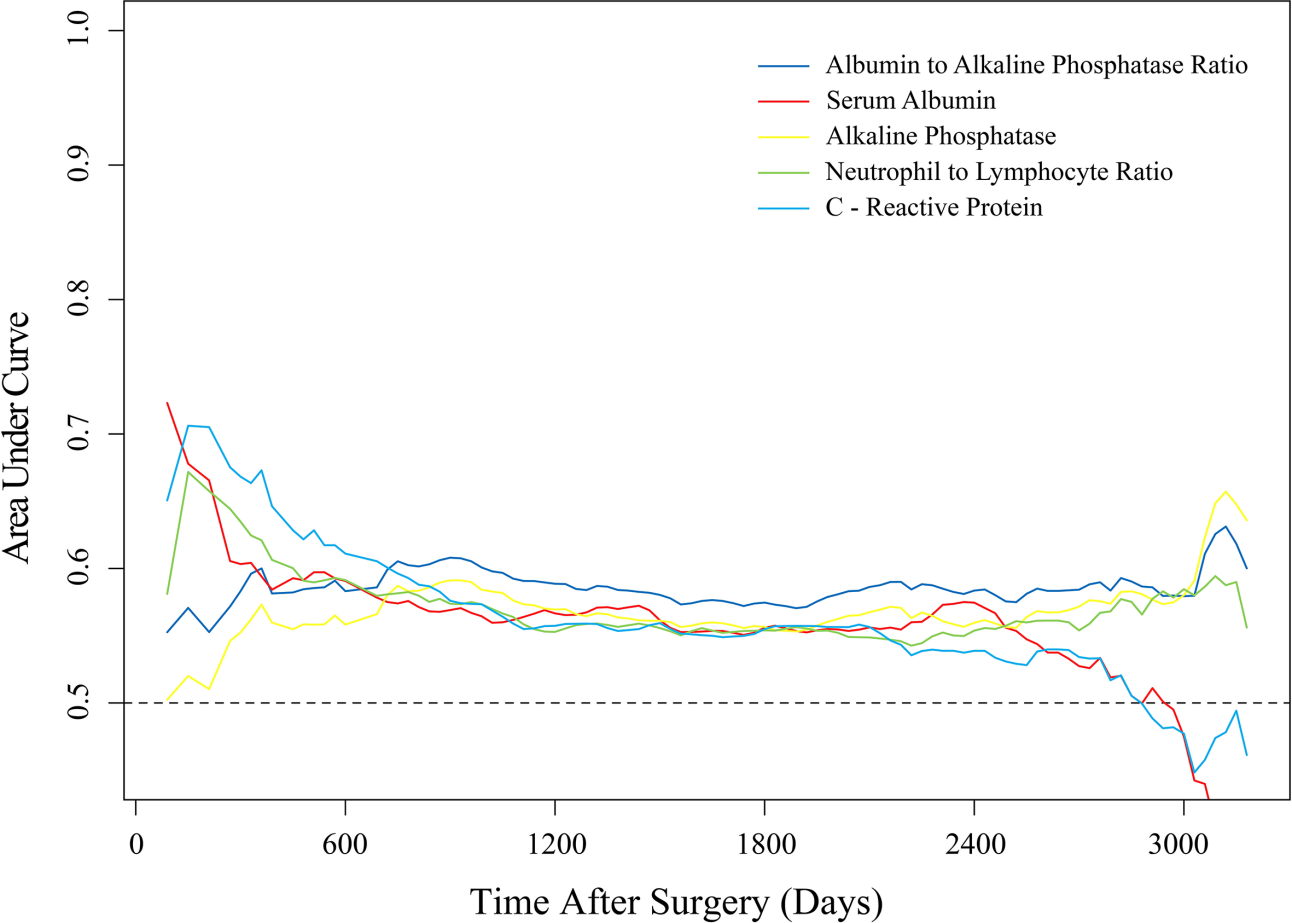
Time-dependent ROC analysis of the discriminatory ability of laboratory-assayed biomarkers for postoperative survival during follow-up.

**Figure 3** shows the dynamic AUCs derived from the t-ROC analysis for all estimated peripheral blood markers. The AUCs for AAPR predicting postoperative survival ranged from 0.57 to 0.63 throughout the follow-up assessment, especially after ≥ 2 years at follow-up, showing a better discriminatory capacity than CRP and NLR. AAPR had significant predictive roles regarding 1-year OS, 3-year OS, 5-year OS, and 10-year OS with AUCs of 0.60, 0.60, 0.58, and 0.62, respectively (*P*<0.050).

### 3.3 Comparison Between AAPR Groups in the Entire Cohort

#### 3.3.1 Preoperative AAPR and Patient Characteristics

**Table 1** exhibits demographic differences in perioperative characteristics between the two AAPR groups. We found that patients with AAPR<0.50 had a significantly lower BMI (*P*=0.003), significantly larger tumor size (*P*<0.001), and a significantly higher percentage of perineural/neural invasion compared with patients with AAPR≥0.50. There were no significant differences in other clinicopathological features between the two AAPR groups.

#### 3.3.2 Preoperative AAPR and In-Hospital Outcomes

Postoperative complication rate was found to be significantly higher in patients with AAPR<0.50 than in those with AAPR≥0.50 (25.6% vs. 16.1%; *P*=0.035; **Table 1**). There was no significant difference in 30-day mortality (0% vs. 0.7%; *P*=1.0) or 90-day mortality (5.8% vs. 1.9%; *P*=0.095) between the two AAPR groups (**Table 1**).

#### 3.3.3 Preoperative AAPR and Survival Outcomes

**Figure 4:**
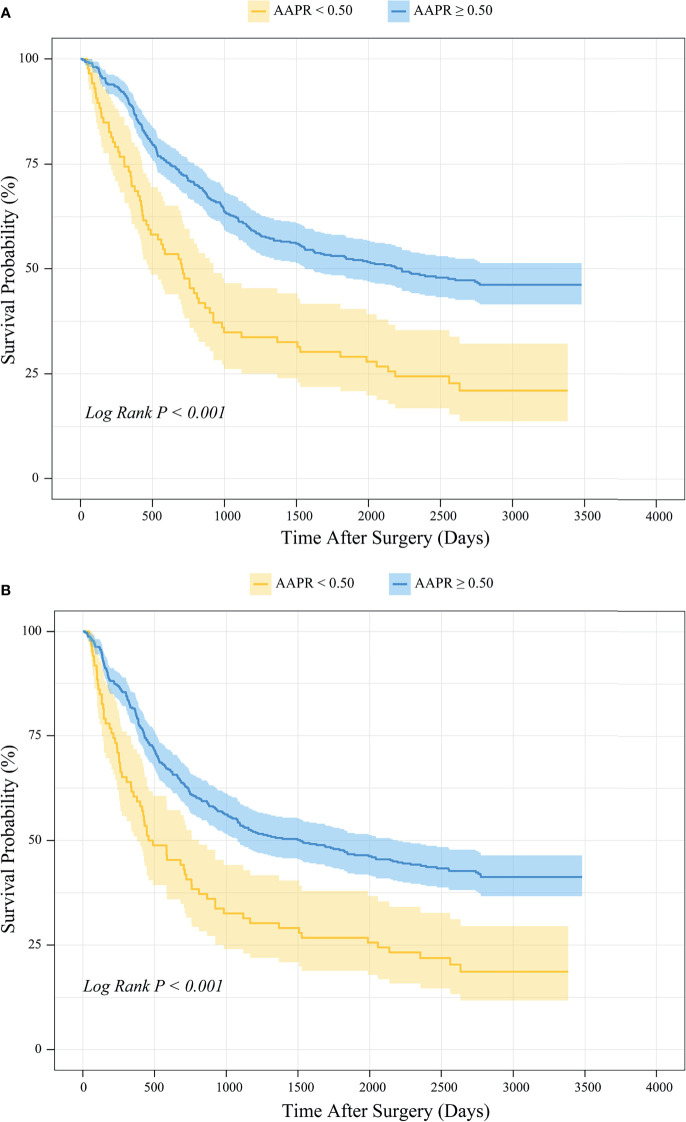
Kaplan-Meier survival analysis of **(A)** OS and **(B)** PFS between patients stratified by preoperative AAPR.

Kaplan-Meier survival analysis revealed that the mean OS time estimates for the AAPR<0.50 and AAPR≥0.50 groups were 1267 ± 134 (95% CI: 1004-1530) days and 2082 ± 68 (95% CI: 1948-2216) days, respectively. Patients with AAPR<0.50 and with AAPR≥0.50 had an OS rate of 22.1% and 47.0% after follow-up, respectively. In addition, the mean PFS time estimates for the AAPR<0.50 and AAPR≥0.50 groups were 1138 ± 133 (95% CI: 877-1399) days and 1877 ± 71 (95% CI: 1737-2016) days, respectively. The PFS rates after follow-up for patients with AAPR<0.50 and with AAPR≥0.50 were 19.8% and 42.1%. Thus, both OS and PFS were significantly shorter in patients with AAPR<0.50 than those with AAPR≥0.50 (Log-rank *P*<0.001; **Figures 4A, B**).

#### 3.3.4 Univariable and Multivariable Analysis on Prognostic Factors of OS

Univariable Cox proportional hazards regression analysis based on the entire patient cohort showed that for each additional unit of age (*P*<0.001), BMI (*P*=0.010), NLR (*P*=0.008) and tumor size (*P*<0.001), male population (*P*=0.025), cardiovascular comorbidity (*P*=0.024), AAPR<0.50 (*P*<0.001), vascular invasion (*P*=0.001), lymphatic invasion (*P*=0.001), T_3_ stage tumor (*P*<0.001), LNM (*P*<0.001), adjuvant chemotherapy (*P*<0.001), and adjuvant radiotherapy (*P*=0.024) were significantly associated with postoperative OS, as shown in **Table 2**.

**Table 2:** Univariable and multivariable analyses of the prognostic factors in the entire cohort.

Characteristics	Overall Survival				Progression Free Survival			
	Univariable Analysis		Multivariable Analysis		Univariable Analysis		Multivariable Analysis	
	HR with 95%CI	*P* Value	HR with 95%CI	*P* Value	HR with 95%CI	*P* Value	HR with 95%CI	*P* Value
Age (Per 1 Year Increase)	1.025 (1.012 - 1.038)	<0.001	1.037 (1.022 - 1.052)	<0.001	1.014 (1.002 - 1.026)	0.023	1.027 (1.013 - 1.041)	<0.001
Gender								
Female	Reference				Reference			
Male	1.46 (1.05 - 2.02)	0.025	1.25 (0.89 - 1.76)	0.19	1.40 (1.03 - 1.91)	0.033	1.22 (0.88 - 1.68)	0.23
Body Mass Index (Per 1 kg/m^2^ Increase)	0.949 (0.912 - 0.987)	0.010	0.950 (0.910 - 0.992)	0.020	0.953 (0.917 - 0.990)	0.013	0.943 (0.904 - 0.983)	0.006
Gastrointestinal Comorbidity								
Absent	Reference				Reference			
Present	1.11 (0.69 - 1.79)	0.66			1.08 (0.68 - 1.71)	0.75		
Cardio-Cerebrovascular Comorbidity								
Absent	Reference				Reference			
Present	1.43 (1.05 - 1.96)	0.024	1.21 (0.87 - 1.70)	0.26	1.30 (0.96 - 1.77)	0.095	1.28 (0.92 - 1.77)	0.14
Diabetes Mellitus								
Absent	Reference				Reference			
Present	0.91 (0.43 - 1.92)	0.80			0.97 (0.48 - 1.95)	0.92		
Respiratory Comorbidity								
Absent	Reference				Reference			
Present	1.10 (0.63 - 1.91)	0.74			0.93 (0.53 - 1.62)	0.79		
Hepatobiliary Comorbidity								
Absent	Reference				Reference			
Present	1.19 (0.68 - 2.07)	0.54			1.15 (0.66 - 2.01)	0.62		
Neutrophil to Lymphocyte Ratio (Per Unit Increase)	1.090 (1.023 - 1.16)	0.008	1.053 (0.969 - 1.144)	0.22	1.062 (0.997 - 1.131)	0.061	0.991 (0.917 - 1.071)	0.82
C - Reactive Protein (Per 1 mg/L Increase)	1.007 (0.999 - 1.016)	0.11	0.990 (0.978 - 1.001)	0.082	1.005 (0.997 - 1.014)	0.22		
Albumin to Alkaline Phosphatase Ratio								
≥ 0.50	Reference				Reference			
< 0.50	2.11 (1.60 - 2.78)	<0.001	1.92 (1.44 - 2.54)	<0.001	1.93 (1.48 - 2.53)	<0.001	1.66 (1.26 - 2.18)	< 0.001
Differentiation Grade								
Well - Moderate	Reference				Reference			
Poor	1.13 (0.89 - 1.45)	0.32			1.16 (0.92 - 1.47)	0.21		
Vascular Invasion								
Absent	Reference				Reference			
Present	1.92 (1.32 - 2.79)	0.001	1.66 (0.61 - 4.55)	0.32	1.90 (1.31 - 2.74)	0.001	2.15 (0.87 - 5.30)	0.096
Lymphatic Invasion								
Absent	Reference				Reference			
Present	2.02 (1.36 - 3.01)	0.001	1.10 (0.37 - 3.25)	0.86	1.85 (1.24 - 2.74)	0.002	0.72 (0.27 - 1.92)	0.51
Perineurium & Neural Invasion								
Absent	Reference				Reference			
Present	1.48 (0.94 - 2.34)	0.091	1.00 (0.60 - 1.66)	1.0	1.39 (0.89 - 2.17)	0.14	1.10 (0.68 - 1.79)	0.69
Tumor Size (Per 0.5 cm Increase)	1.160 (1.082 - 1.244)	<0.001	1.090 (0.999 - 1.189)	0.054	1.160 (1.085 - 1.241)	<0.001	1.101 (1.016 - 1.194)	0.019
T Stage								
T_1_ - T_2_	Reference				Reference			
T_3_	1.70 (1.29 - 2.24)	<0.001	1.39 (1.04 - 1.85)	0.027	1.59 (1.23 - 2.07)	<0.001	1.23 (0.93 - 1.62)	0.15
N Stage								
N_0_	Reference				Reference			
N_1_ - N_3_	2.83 (2.21 - 3.64)	<0.001	2.44 (1.85 - 3.22)	< 0.001	2.72 (2.14 - 3.46)	<0.001	2.06 (1.59 - 2.68)	< 0.001
TNM Stage								
I - II	Reference				Reference			
III	1.44 (0.89 - 1.47)	0.29			1.17 (0.93 - 1.49)	0.19		
Adjuvant Chemotherapy								
Not Received	Reference				Reference			
Received	1.59 (1.26 - 2.01)	<0.001	1.39 (1.04 - 1.85)	0.024	1.94 (1.55 - 2.44)	<0.001	1.64 (1.25 - 2.16)	< 0.001
Adjuvant Radiotherapy								
Not Received	Reference				Reference			
Received	1.46 (1.05 - 2.03)	0.024	1.05 (0.73 - 1.50)	0.79	2.13 (1.57 - 2.89)	<0.001	1.47 (1.06 - 2.06)	0.022
Postoperative Complications								
Absent	Reference				Reference			
Present	1.33 (0.99 - 1.78)	0.056	1.42 (1.04 - 1.92)	0.025	1.15 (0.86 - 1.54)	0.33		

CI, Confidence Interval; HR, Hazard Ratio.

After adjustment by all covariable estimates holding *P*<0.15, multivariable Cox proportional hazards regression analysis showed that for every 1 year increase in age (HR: 1.037; 95% CI: 1.022-1.052; *P*<0.001), every 1 kg/m^2^ increase in BMI (HR: 0.950; 95% CI: 0.910-0.992; *P*=0.020), AAPR<0.50 (HR: 1.92; 95% CI: 1.44-2.54; *P*<0.001), T_3_ stage tumor (HR: 1.39; 95% CI: 1.04-1.85; *P*=0.027), LNM (HR: 2.44; 95% CI: 1.85-3.22; *P*<0.001), adjuvant chemotherapy (HR: 1.39; 95% CI: 1.04-1.85; *P*=0.024), and the occurrence of postoperative complications (HR: 1.42; 95% CI: 1.04-1.92; *P*=0.025) could be independent prognostic factors for OS in ESCC patients undergoing esophagectomy (**Table 2**).

**Figure 5:**
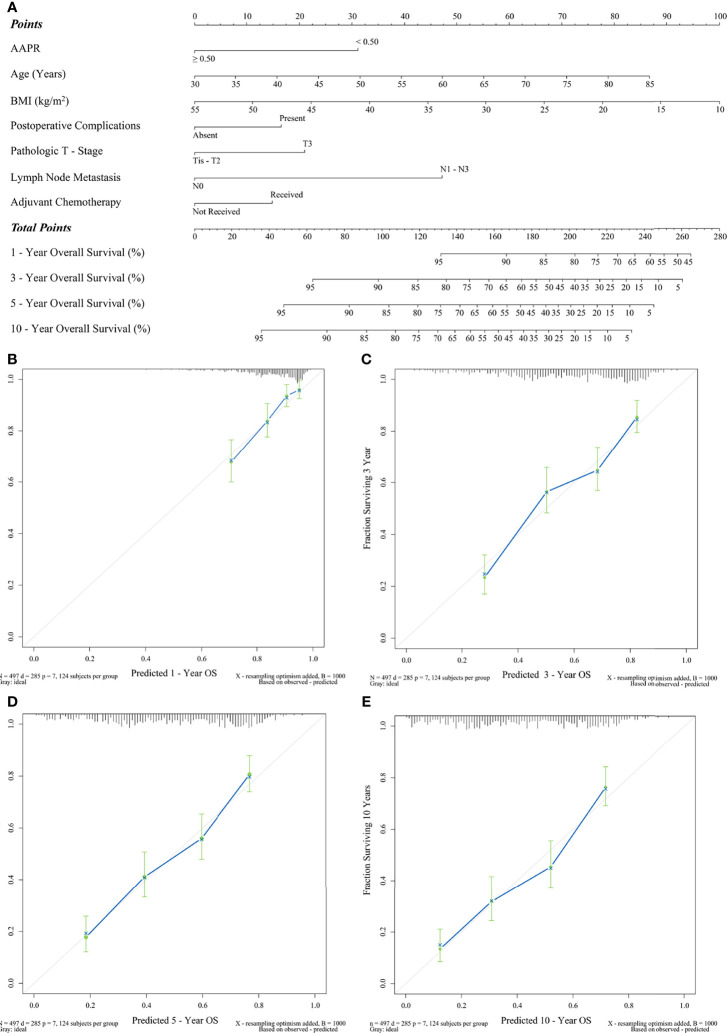
Prediction of OS by preoperative AAPR. **(A)** AAPR composite nomogram and its calibration curves **(B)** 1-year OS, **(C)** 3-year OS, **(D)** 5-year OS, and **(E)** 10-year OS.

Finally, we constructed a prognostic nomogram incorporating all the above independent indicators to represent a visual and comprehensive risk evaluation scale for predicting postoperative OS, as shown in **Figure 5A**. We found that both LNM and AAPR were the most predominant predictors of postoperative OS in ESCC patients (*P*<0.001). This nomogram based on AAPR and other independent predictors presented a significant contribution to the discrimination of OS probability with a C-statistic of 0.71 (95% CI: 0.62-0.80; *P*<0.001). **Figures 5B-E** are calibration curves assessing the predictive strength of our nomogram for OS probability. These curves showed a significant agreement between the OS predicted by our nomogram and the real-world OS at different follow-up periods.

#### 3.3.5 Univariable and Multivariable Analysis on Prognostic Factors of PFS

Univariable Cox proportional hazards regression analysis based on the entire patient cohort showed that for each additional unit of age (*P*=0.023), BMI (*P*=0.013) and tumor size (*P*<0.001), male population (*P*=0.033), AAPR<0.50 (*P*<0.001), vascular invasion (*P*=0.001), lymphatic invasion (*P*=0.002), T_3_ stage tumor (*P*<0.001), LNM (*P*<0.001), adjuvant chemotherapy (*P*<0.001), and adjuvant radiotherapy (*P*<0.001) were significantly associated with postoperative PFS (**Table 2**).

Multivariable Cox proportional hazards regression models built on clinicopathological parameters showing *P*<0.15 further demonstrated that for every 1 year increase in age (HR: 1.027; 95% CI: 1.013-1.041; *P*<0.001), every 1 kg/m^2^ increase in BMI (HR: 0.943; 95% CI: 0.904-0.983; *P*=0.006), every 0.5 cm increase in tumor size (HR: 1.101; 95% CI: 1.016-1.194; *P*=0.019), AAPR<0.50 (HR: 1.66; 95% CI: 1.25-2.18; *P*<0.001), LNM (HR: 2.06; 95% CI: 1.59-2.68; *P*<0.001), adjuvant chemotherapy (HR: 1.64; 95% CI: 1.25-2.16; *P*<0.001), and adjuvant radiotherapy (HR: 1.47; 95% CI: 1.06-2.06; *P*=0.022) were determined to be independent predictors of PFS in surgically resected ESCC (**Table 2**).

**Figure 6:**
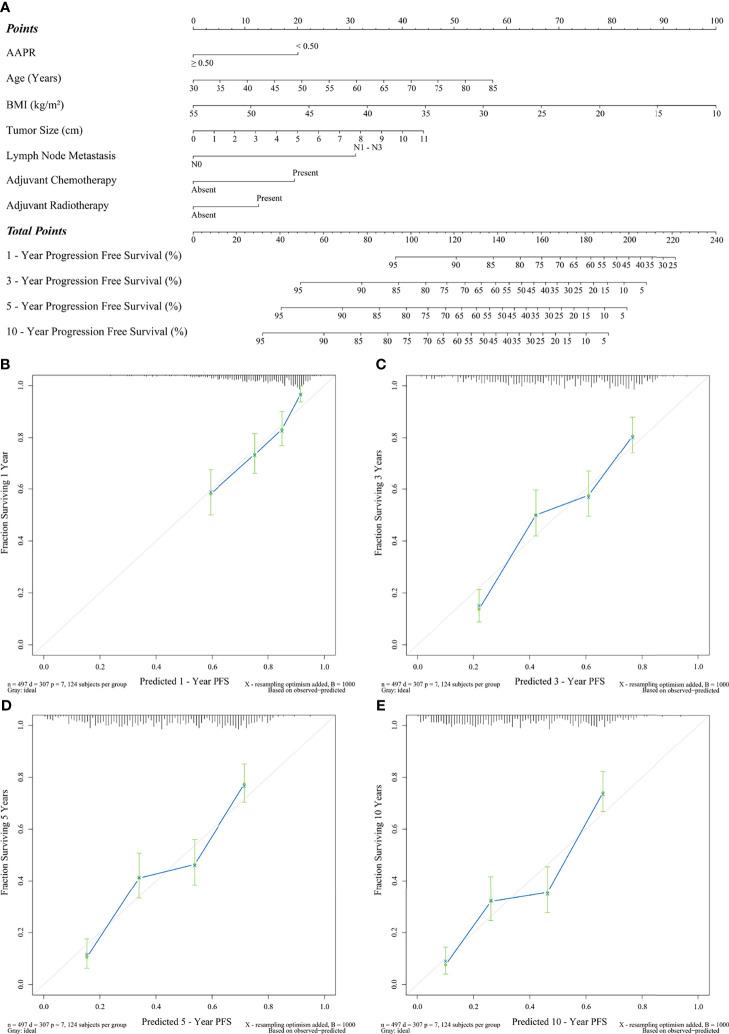
Prediction of PFS by preoperative AAPR. **(A)** AAPR composite nomogram and its calibration curves **(B)** 1-year PFS, **(C)** 3-year PFS, **(D)** 5-year PFS, and **(E)** 10-year PFS.

Finally, **Figure 6A** indicates a prognostic nomogram integrated by all the independent predictors of PFS mentioned above. LNM, AAPR, and adjuvant chemotherapy were the prognostic indicators contributing the most to postoperative PFS (*P*<0.001). The C-statistic of this nomogram was 0.70 (95% CI: 0.61-0.78), which had statistical significance when applied to differentiate the probability of PFS (*P*<0.001). In addition, as shown in **Figures 6B-E**, all calibration curves also showed a significant agreement between the nomogram-predicted PFS and the real-world PFS at different follow-up periods.

### 3.4 Subgroup Analysis on the Prognostic Value of AAPR

**Figure 7:**
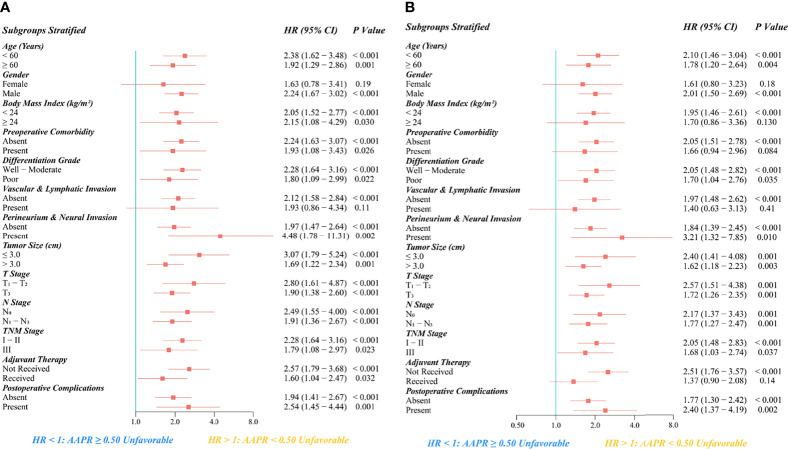
Subgroup analysis regarding the prognostic significance of preoperative AAPR on **(A)** OS and **(B)** PFS after esophagectomy for ESCC.

As the subgroup survival results indicated, an unfavorable prognostic value of AAPR<0.50 for OS and PFS remained significantly reliable across all subgroups stratified by age, grade of differentiation, presence of perineural and neural invasion, tumor size, T stage, N stage, TNM stage, and occurrence of postoperative complications (**Figures 7A, B**).

In addition, AAPR<0.50 was significantly associated with more inferior OS in all subgroups of BMI, preoperative comorbidity, adjuvant therapy, and male patients without vascular or lymphatic invasion. Moreover, AAPR<0.50 was also significantly associated with more inferior PFS in the subgroups of male gender, BMI <24 kg/m^2^, absence of preoperative comorbidity, absence of vascular or lymphatic invasion, and patients not receiving adjuvant therapy.

### 3.5 Comparison Between AAPR Groups in the PSM Cohort

#### 3.5.1 PSM Cohort Generation

**Figure 8:**
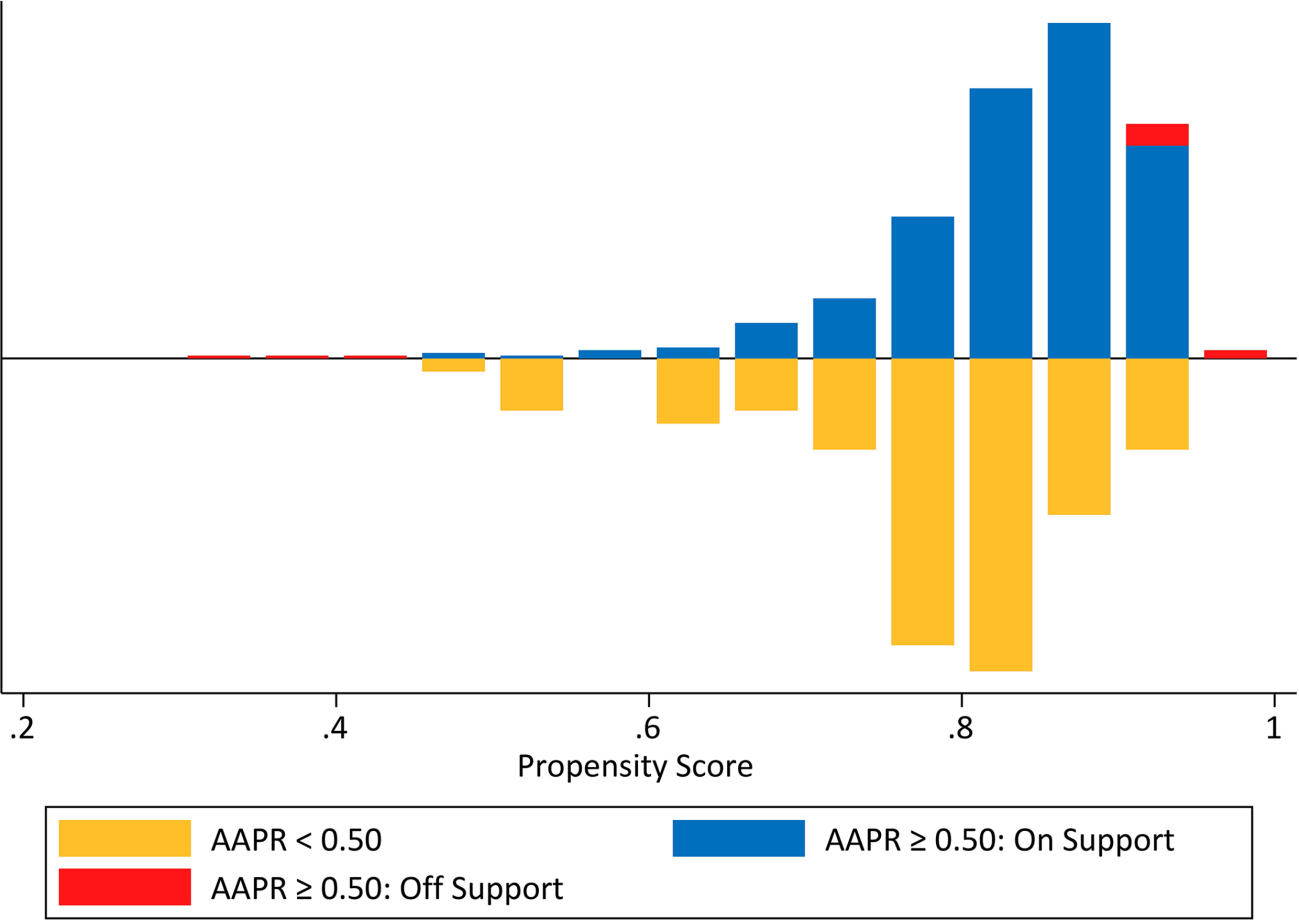
Mirrored histogram presenting the propensity score distribution and the overlap between unmatched and matched samples in the two AAPR groups.

We determined BMI, NLR, CRP, perineural and neural invasion, and tumor size, all of which differed significantly between the two AAPR groups and were further engaged in the PSM procedure (**Figure 8**). As a result, this PSM analysis yielded 75 fully matched pairs of patients grouped by preoperative AAPR, as shown in **Table 3**.

**Table 3:** Patient characteristics and in-hospital outcomes of the propensity score matched cohort.

Characteristics	Propensity Score Matched Cohort			*P* Value
	Total (*N* = 150)	AAPR ≥ 0.50 (*N* = 75)	AAPR < 0.50 (*N* = 75)	
Age (Years)				
Mean ± SD	59.61 ± 9.39	59.81 ± 10.05	59.40 ± 8.74	0.63
Median (IQR)	60 (54 - 66)	60 (53 - 68)	58 (54 - 66)	
Gender (n, %)				
Female	25 (16.7%)	11 (14.7%)	14 (18.7%)	0.51
Male	125 (83.3%)	64 (85.3%)	61 (81.3%)	
Body Mass Index (kg/m^2^)				
Mean ± SD	20.70 ± 2.84	20.52 ± 2.75	20.88 ± 2.94	0.58
Median (IQR)	20.24 (18.50 - 22.66)	20.08 (18.49 - 22.66)	20.38 (18.51 - 22.84)	
Gastrointestinal Comorbidity (n, %)				
Absent	144 (96.0%)	72 (96.0%)	72 (96.0%)	1.0
Present	6 (4.0%)	3 (4.0%)	3 (4.0%)	
Cardio-Cerebrovascular Comorbidity (n, %)				
Absent	127 (84.7%)	61 (81.3%)	66 (88.0%)	0.26
Present	23 (15.3%)	14 (18.7%)	9 (12.0%)	
Diabetes Mellitus (n, %)				
Absent	146 (97.3%)	73 (97.3%)	73 (97.3%)	1.0
Present	4 (2.7%)	2 (2.7%)	2 (2.7%)	
Respiratory Comorbidity (n, %)				
Absent	145 (96.7%)	72 (96.0%)	73 (97.3%)	1.0
Present	5 (3.3%)	3 (4.0%)	2 (2.7%)	
Hepatobiliary Comorbidity (n, %)				
Absent	146 (97.3%)	73 (97.3%)	73 (97.3%)	1.0
Present	4 (2.7%)	2 (2.7%)	2 (2.7%)	
Neutrophil to Lymphocyte Ratio				
Mean ± SD	2.95 ± 1.45	2.99 ± 1.47	2.91 ± 1.43	0.76
Median (IQR)	2.62 (1.86 - 3.81)	2.73 (1.89 - 3.79)	2.56 (1.70 - 3.91)	
C - Reactive Protein (mg/L)				
Mean ± SD	7.98 ± 11.13	7.81 ± 11.96	8.16 ± 10.32	0.080
Median (IQR)	3.54 (1.47 - 8.89)	2.78 (1.02 - 10.58)	4.39 (2.22 - 8.79)	
AAPR Value				
Mean ± SD	0.55 ± 0.15	0.67 ± 0.13	0.44 ± 0.05	< 0.001
Median (IQR)	0.50 (0.46 - 0.63)	0.62 (0.57 - 0.76)	0.46 (0.41 - 0.48)	
Differentiation Grade (n, %)				
Well	39 (26.0%)	20 (26.7%)	19 (25.3%)	0.88
Moderate	71 (47.3%)	34 (45.3%)	37 (49.3%)	
Poor	40 (26.7%)	21 (28.0%)	19 (25.3%)	
Vascular Invasion (n, %)				
Absent	136 (90.7%)	68 (90.7%)	68 (90.7%)	1.0
Present	14 (9.3%)	7 (9.3%)	7 (9.3%)	
Lymphatic Invasion (n, %)				
Absent	138 (92.0%)	69 (92.0%)	69 (92.0%)	1.0
Present	12 (8.0%)	6 (8.0%)	6 (8.0%)	
Perineurium & Neural Invasion (n, %)				
Absent	136 (90.7%)	68 (90.7%)	68 (90.7%)	1.0
Present	14 (9.3%)	7 (9.3%)	7 (9.3%)	
Tumor Size (cm)				
Mean ± SD	4.27 ± 1.68	4.26 ± 1.85	4.28 ± 1.51	0.94
Median (IQR)	4.0 (3.0 - 5.0)	4.0 (3.0 - 5.0)	4.0 (3.0 - 5.0)	
T Stage (n, %)				
T_1_	12 (8.0%)	7 (9.3%)	5 (6.7%)	0.71
T_2_	27 (18.0%)	12 (16.0%)	15 (20.0%)	
T_3_	111 (74.0%)	56 (74.7%)	55 (73.3%)	
N Stage (n, %)				
N_0_	73 (48.7%)	41 (54.7%)	32 (42.7%)	0.39
N_1_	65 (43.3%)	30 (40.0%)	35 (46.7%)	
N_2_	7 (4.7%)	2 (2.7%)	5 (6.7%)	
N_3_	5 (3.3%)	2 (2.7%)	3 (4.0%)	
TNM Stage (n, %)				
I	34 (22.7%)	17 (22.7%)	17 (22.7%)	0.84
II	75 (50.0%)	36 (48.0%)	39 (52.0%)	
III	41 (27.3%)	22 (29.3%)	19 (25.3%)	
Adjuvant Chemotherapy (n, %)				
Not Received	103 (68.7%)	54 (72.0%)	49 (65.3%)	0.38
Received	47 (31.3%)	21 (28.0%)	26 (34.7%)	
Adjuvant Radiotherapy (n, %)				
Not Received	133 (88.7%)	68 (90.7%)	65 (86.7%)	0.44
Received	17 (11.3%)	7 (9.3%)	10 (13.3%)	
Postoperative Complications (n, %)				
Absent	113 (75.3%)	58 (77.3%)	55 (73.3%)	0.57
Present	37 (24.7%)	17 (22.7%)	20 (26.7%)	
30 - Day Mortality (n, %)				
Absent	148 (98.7%)	74 (98.7%)	74 (98.7%)	1.0
Present	2 (1.3%)	1 (1.3%)	1 (1.3%)	
90 - Day Mortality (n, %)				
Absent	143 (95.3%)	73 (97.3%)	70 (93.3%)	0.44
Present	7 (4.7%)	2 (2.7%)	5 (6.7%)	

AAPR, Albumin to Alkaline Phosphatase Ratio; IQR, Interquartile Range; SD, Standard Deviation.

#### 3.5.2 Preoperative AAPR and In-Hospital Outcomes

In the PSM cohort, there was no significant difference between patients with AAPR<0.50 and those with AAPR≥0.50 in terms of postoperative complication rate (26.7% vs. 22.7%; *P*=0.57), 30-day mortality rate (1.3% vs. 1.3%; *P*=1.0), or 90-day mortality rate (6.7% vs. 2.7%; *P*=0.44) (**Table 3**).

#### 3.5.3 Preoperative AAPR and Survival Outcomes

**Figure 9:**
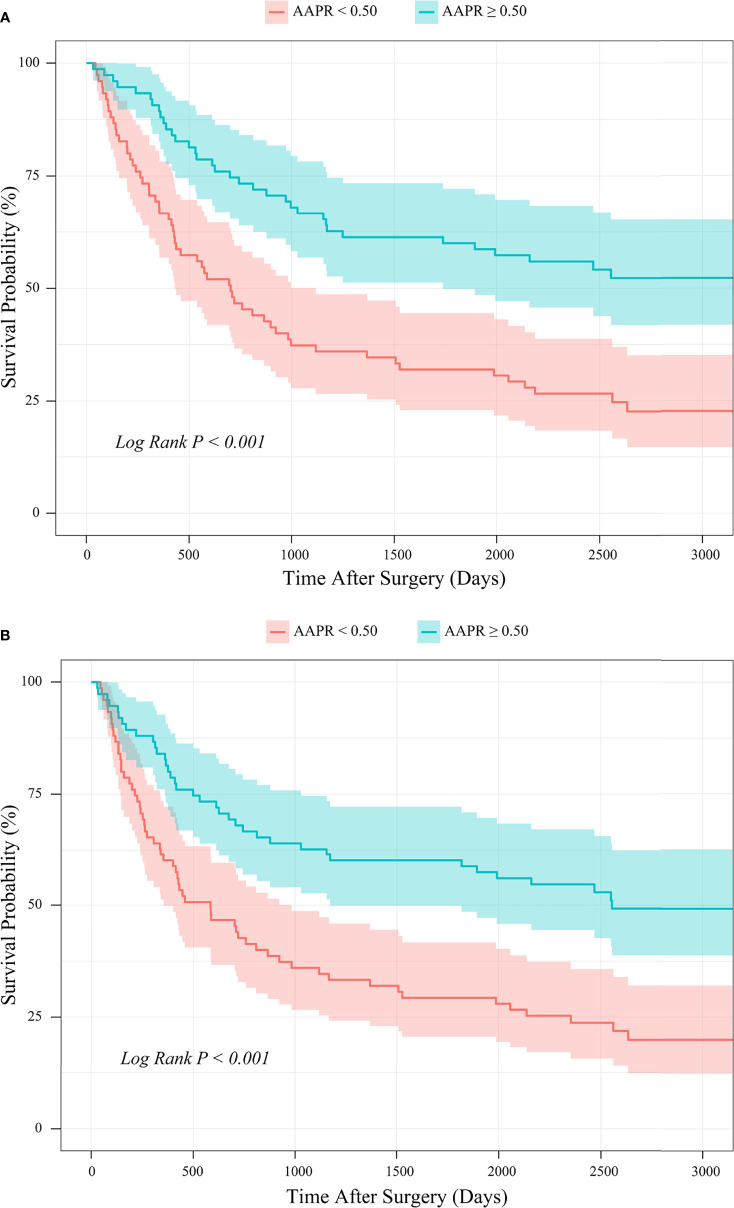
PSM-based Kaplan-Meier survival analysis of **(A)** OS and **(B)** PFS between patient groups stratified by preoperative AAPR.

Kaplan-Meier survival analysis based on the PSM cohort showed that the mean OS time estimates for the AAPR<0.50 and AAPR≥0.50 groups were 1308 ± 148 (95% CI: 1017-1598) days and 2219 ± 157 (95% CI: 1911-2527) days, respectively. Patients with AAPR<0.50 and with AAPR≥0.50 had an OS rate of 24.0% and 53.3% after follow-up. In addition, the mean PFS time estimates for the AAPR<0.50 and AAPR≥0.50 groups were 1203 ± 146 (95% CI: 917-1489) days and 2112 ± 164 (95% CI: 1791-2434) days, respectively. The PFS rates after follow-up for patients with AAPR<0.50 and with AAPR≥0.50 were 21.3% and 50.7%, respectively. Thus, in the PSM cohort, patients with AAPR<0.50 had significantly worse outcomes of both OS and PFS than those with AAPR≥0.50 (Log-rank *P*<0.001; **Figures 9A, B**).

#### 3.5.4 Univariable and Multivariable Analysis on Prognostic Factors of OS

Univariable Cox proportional hazards regression analysis revealed that for every 1 year increase in age (*P*=0.013), AAPR<0.50 (*P*<0.001), LNM (*P*<0.001), and the occurrence of postoperative complications (*P*=0.017) were significantly associated with shortened OS of ESCC patients in the PSM cohort (**Table 4**). Subsequently, in multivariable Cox proportional hazards regression analysis, all of them were further identified as independent prognostic biomarkers of postoperative OS after adjustment for other clinicopathological characteristics with *P*<0.15. In addition, LNM (HR: 2.56; 95% CI: 1.66-3.94; *P*<0.001) and AAPR<0.50 (HR: 2.40; 95% CI: 1.56-3.69; *P*<0.001) showed the most substantial prognostic significance associated with unfavorable OS in surgically resected ESCC (**Table 4**). A multivariable Cox proportional hazards regression model consisting of AAPR and other independent prognostic predictors had a significant discriminatory power for OS probability as indicated by a C-statistic of 0.71 (95% CI: 0.57-0.86; *P*<0.001).

**Table 4:** Univariable and multivariable analyses of the prognostic factors in the propensity score matched cohort.

Characteristics	Overall Survival				Progression Free Survival			
	Univariable Analysis		Multivariable Analysis		Univariable Analysis		Multivariable Analysis	
	HR with 95%CI	*P* Value	HR with 95%CI	*P* Value	HR with 95%CI	*P* Value	HR with 95%CI	*P* Value
Age (Per 1 Year Increase)	1.028 (1.006 - 1.051)	0.013	1.028 (1.005 - 1.051)	0.018	1.026 (1.005 - 1.048)	0.017	1.029 (1.004 - 1.054)	0.020
Gender								
Female	Reference				Reference			
Male	1.06 (0.61 - 1.85)	0.83			1.01 (0.59 - 1.73)	0.97		
Body Mass Index (Per 1 kg/m^2^ Increase)	0.945 (0.877 - 1.019)	0.14	0.926 (0.855 - 1.003)	0.060	0.947 (0.880 - 1.019)	0.140	0.923 (0.852 - 0.999)	0.048
Gastrointestinal Comorbidity								
Absent	Reference				Reference			
Present	1.24 (0.45 - 3.37)	0.68			1.18 (0.43 - 3.21)	0.75		
Cardio-Cerebrovascular Comorbidity								
Absent	Reference				Reference			
Present	1.29 (0.74 - 2.25)	0.37			1.25 (0.72 - 2.17)	0.43		
Diabetes Mellitus								
Absent	Reference				Reference			
Present	0.37 (0.05 - 2.62)	0.32			0.35 (0.05 - 2.49)	0.29		
Respiratory Comorbidity								
Absent	Reference				Reference			
Present	1.09 (0.34 - 3.43)	0.89			0.96 (0.30 - 3.03)	0.94		
Hepatobiliary Comorbidity								
Absent	Reference				Reference			
Present	1.26 (0.40 - 3.98)	0.70			1.12 (0.35 - 3.53)	0.85		
Neutrophil to Lymphocyte Ratio (Per Unit Increase)	1.094 (0.943 - 1.248)	0.26			1.058 (0.920 - 1.216)	0.43		
C - Reactive Protein (Per 1 mg/L Increase)	1.000 (0.982 - 1.019)	0.99			1.004 (0.986 - 1.021)	0.68		
Albumin to Alkaline Phosphatase Ratio								
≥ 0.50	Reference				Reference			
< 0.50	2.34 (1.53 - 3.57)	<0.001	2.40 (1.56 - 3.69)	<0.001	2.26 (1.49 - 3.42)	<0.001	2.30 (1.50 - 3.53)	<0.001
Differentiation Grade								
Well - Moderate	Reference				Reference			
Poor	1.16 (0.74 - 1.82)	0.51			1.32 (0.86 - 2.03)	0.21		
Vascular Invasion								
Absent	Reference				Reference			
Present	1.35 (0.70 - 2.61)	0.37			1.31 (0.68 - 2.52)	0.42		
Lymphatic Invasion								
Absent	Reference				Reference			
Present	1.08 (0.52 - 2.24)	0.83			1.03 (0.50 - 2.12)	0.94		
Perineurium & Neural Invasion								
Absent	Reference				Reference			
Present	1.26 (0.63 - 2.51)	0.52			1.17 (0.59 - 2.33)	0.66		
Tumor Size (Per 0.5 cm Increase)	1.050 (0.934 - 1.180)	0.41			1.084 (0.967 - 1.216)	0.17		
T Stage								
T_1_ - T_2_	Reference				Reference			
T_3_	1.34 (0.83 - 2.16)	0.23			1.19 (0.76 - 1.88)	0.45		
N Stage								
N_0_	Reference				Reference			
N_1_ - N_3_	2.60 (1.69 - 3.99)	<0.001	2.56 (1.66 - 3.94)	<0.001	2.66 (1.75 - 4.04)	<0.001	2.47 (1.57 - 3.89)	<0.001
TNM Stage								
I - II	Reference				Reference			
III	1.17 (0.75 - 1.82)	0.49			1.32 (0.86 - 2.02)	0.21		
Adjuvant Chemotherapy								
Not Received	Reference				Reference			
Received	1.27 (0.82 - 1.95)	0.28			1.44 (0.95 - 2.19)	0.087	1.25 (0.76 - 2.06)	0.38
Adjuvant Radiotherapy								
Not Received	Reference				Reference			
Received	1.20 (0.66 - 2.15)	0.55			1.73 (1.00 - 3.02)	0.052	1.02 (0.55 - 1.88)	0.96
Postoperative Complications								
Absent	Reference				Reference			
Present	1.72 (1.10 - 2.69)	0.017	1.62 (1.03 - 2.55)	0.036	1.58 (1.02 - 2.45)	0.042	1.43 (0.90 - 2.25)	0.13

CI, Confidence Interval; HR, Hazard Ratio.

#### 3.5.5 Univariable and Multivariable Analysis on Prognostic Factors of PFS

In univariable Cox proportional hazards regression analysis, we found that for every 1 year increase in age (*P*=0.017), AAPR<0.50 (*P*<0.001), LNM (*P*<0.001), and the occurrence of postoperative complications (*P*=0.042) were significantly associated with worsening PFS of ESCC patients in the PSM cohort (**Table 4**). Multivariable Cox proportional hazards regression model on clinicopathological covariables with *P*<0.15 further suggested that for every 1 year increase in age (HR: 1.029; 95% CI: 1.004-1.054; *P*=0.020), every 1 kg/m^2^ increase in BMI (HR: 0.923; 95% CI: 0.852-0.999; *P*=0.048), AAPR<0.50 (HR: 2.30; 95% CI: 1.50-3.53; *P*<0.001), and LNM (HR: 2.47; 95% CI: 1.57-3.89; *P*<0.001) could be regarded as independent predictors of postoperative PFS (**Table 4**). Finally, the multivariable Cox proportional hazards regression model involving AAPR and other independent prognostic predictors had a significant discriminatory power for the probability of PFS with a C-statistic of 0.71 (95% CI: 0.56-0.86; *P*<0.001).

## 4 Discussion

### 4.1 Key Results and Interpretations

Since Chan et al. (16) first introduced this simply synthetic biomarker based on sALB and serum ALP for estimating the outcome of the procedure in hepatocellular carcinoma, AAPR has been widely demonstrated to be promisingly indicative of post-treatment prognosis in a series of malignant tumors, including nasopharyngeal carcinoma (17), urothelial carcinoma (18), cholangiocarcinoma (19), lung cancer (12, 20), bladder cancer (21), and pancreatic cancer (22, 23). Several recent evidence-based literature reviews have also provided comprehensive results suggesting a potent prognostic impact of AAPR in human solid tumors, regardless of their histological origins (15, 24). However, until recently, there was no investigation aimed at the clinical significance of AAPR in surgically resected ESCC.

To our knowledge, our study was the first to employ a PSM analysis to elucidate potential prognostic effects of preoperative AAPR on OS and PFS in surgically resected ESCC. An AAPR of 0.50 was considered to be the threshold for prognostic outcome stratification of ESCC patients in our cohort. Patients with AAPR<0.50 had a significantly lower BMI, significantly larger tumor size, significantly higher NLR and CRP, and a significantly higher proportion of perineural and neural invasion than patients with AAPR≥0.50. Similar to previous reports in surgical oncology, we found that a decreased level of preoperative AAPR was significantly associated with poorer survival outcomes following esophagectomy for cancer. In our study, by applying an optimal cutoff point of 0.50, AAPR could play a considerable role in the effective risk stratification of OS and PFS in surgical patients with ESCC. In addition, we further established two nomograms based on preoperative AAPR with the aim to predict the probability of OS and PFS comprehensively. After calibration, AAPR<0.50 and LNM were finally identified as the two most apparent susceptibility factors for death from any cause or cancer progression in the present cohort of ESCC patients.

One of our study focuses was to determine the prognostic utility of AAPR in patients undergoing esophagectomy for ESCC *via* both Cox proportional hazards regression analysis and PSM analysis. PSM has been widely validated as an excellent statistical alternative that collapsed all potential confounders into a single value that matches each subject between study groups with well-balanced baseline characteristics, thus providing an effective way to circumvent the ontological limitations of classical multivariable regression modeling (8, 10, 12). Given such concerns, a total of 75 well-matched pairs of patients were extracted by reviewing the propensity scores of the two AAPR groups. Finally, AAPR was found to exhibit substantial prognostic impact in the entire cohort, and was also validated as a independent prognostic biomarker for OS and PFS in the PSM cohort.

Another highlight of our study was a comprehensive survival analysis of the prognostic roles of AAPR in a specific set of ESCC patient subgroups. Our findings confirmed that AAPR remained statistically reliable for predicting OS and PFS of ESCC patients in most subgroups stratified by clinicopathological characteristics. Furthermore, we observed no significant difference in either OS or PFS between patients with AAPR<0.50 and with AAPR≥0.50 in both women and patients with vascular or lymphatic invasive tumors. We speculated that the limited sample size of these two subgroups might have weakened the demonstrative power of Cox proportional hazards regression models derived.

The following possible biological mechanisms may help to elucidate the links between AAPR and prognosis of surgically resected ESCC. Firstly, hypoalbuminemia characterized by a decline in sALB has not only been considered as a promising indicator of suppression of host immune-nutritional reserve, but also as a potential diagnostic biomarker for human malignancies (14). Hypoalbuminemia also performs as one of the cardinal markers of cachexia during the carcinomatous progression, which is defined as a multifactorial syndrome characterized by an ongoing skeletal muscle loss that cannot be fully reversed by conventional nutritional support and eventually aggravates a functional impairment according to the Washington and Delphi consensus (29), especially throughout the disease trajectory of ESCC (30). In addition, as a negative acute-phase protein, sALB has also been used to reveal changes in the systemic inflammatory response, as hepatocyte synthesis of sALB can be influenced by the biological activity of pro-inflammatory cytokines. Both malnutrition and inflammation contribute to the development of hypoalbuminemia, which leads to impaired surgical tolerance due to an impaired ability to withstand acute injury (8, 10, 12). At the same time, sALB maintains its physiological properties as an antioxidant transporter. Therefore, hypoalbuminemia may impair antitumor immunity and response to adjuvant therapy, leading to unfavorable post-surgical prognosis (15).

Secondly, serum ALP is a hydrolytic enzyme mainly concentrated in the liver, bone, and kidney that catalyze the hydrolytic processes and phosphate transfer in an alkaline environment. An extensive body of evidence suggest that elevated ALP levels can indicate oxidative stress, which is induced by inflammation together with fast-growing tumors, contributing to the production of reactive oxygen species that damage the DNA, protein, and lipid, and promote active mutagenic metabolism (12, 31). In addition, increased cellular permeability in cancer-related inflammation can also increase intracellular ALP concentration and activity (32). Therefore, serum ALP can be considered a valuable marker of carcinogenesis, cancer cell proliferation and metastasis (29).

### 4.2 Clinical Significance

All our findings provide evidence to support the inclusion of a value of AAPR in routine risk assessment prior to esophagectomy to better distinguish patients who have a higher probability of suffering an unfavorable prognosis. AAPR is directly extrapolated from sALB and serum ALP, both of which can be easily and inexpensively obtained through peripheral chemistry test in the routine clinical practice. In addition, the predictive accuracy of postoperative survival in ESCC patients can be improved by identifying their AAPR levels early before surgery. Thus, more accurate prognostic prediction would thus facilitate the development of more individualized treatment plans to improve anti-inflammatory and nutritional care, enhance the tolerance of surgery, and limit potential adverse events.

### 4.3 Limitations

There were several notable limitations of this study.

First, this study was a retrospective analysis on a single-center prospectively collected database that was not externally validated. Therefore, due to the inherent limitations of retrospective nature, potential selection bias might have still attenuated the demonstrative strength of AAPR as a reliable prognostic marker for surgically resectable ESCC, despite our attempts to conduct a well-designed PSM analysis of the included patients based on our reasonably stringent eligibility criteria. In addition, the limited sample size of a single institution might also have negatively affected the strength of evidence in our study. Therefore, we strongly recommend additional multicenter prospective studies in the future to better control for potential confounding covariables to validate the clinical implication of AAPR in surgically resectable ESCC.

Second, the value of AAPR in this study was a single time-point measure preoperatively. However, AAPR was usually a dynamic serum biomarker which significantly fluctuated over time. It would also be essentially meaningful to explore the changes of AAPR during the follow-up period. Hence, a prospective validating analysis aimed on the dynamic prognostic roles of AAPR in surgically resectable ESCC can be regarded as a crucial area of investigation in the future.

Third, serum ALP comprises a heterogeneous group of isozymes presented only in intestinal, placental, and reproductive tissues, which are substantially different from the tissue-nonspecific types mainly produced by the liver, bone, and kidney (32). Unfortunately, ALP isozyme analysis was not a routine laboratory test at our institution during the study period. We suggest that different assays for the source of ALP may further help to understand possible biological mechanisms underlying the prognostic role of AAPR in ESCC.

Fourth, potential differences in the salvage procedures outside our institution might exist during the disease course between the two AAPR groups, which had probably altered the ESCC prognosis in favor of one group in an unintentional manner.

Finally, there was no consensus on the threshold for AAPR in surgical oncology. In the latest studies, the selective cutoff points for AAPR varied.

## 5 Conclusions

In conclusion, our study suggests that preoperative AAPR can be a promising prognostic predictor of OS and PFS in patients undergoing esophagectomy for ESCC. As a simple, convenient, and noninvasive biomarker from routine biochemical tests, AAPR shows excellent potential to help improve the predictive effectiveness of current risk stratification tools for the prognosis of surgically resected ESCC. As the present study still has some limitations, future multicenter prospective studies are urgently needed to confirm and validate our findings.

## Data Availability Statement

The raw data supporting the conclusions of this article will be made available by the authors, without undue reservation.

## Ethics Statement

The studies involving human participants were reviewed and approved by the Institutional Review Board of Sun Yat-Sen University Cancer Center (No: GZR 2018-120) and performed all relevant procedures compliant with the Declaration of Helsinki. The patients/participants provided their written informed consent to participate in this study.

## Author Contributions

We formally declare that all the authors’ individual contributions are as follows. Conceptualization and Supervision, SL and ZW. Data curation, DC, XZ, JZ, and ZW. Formal analysis and Methodology, SL, WZ, and YL. Funding acquisition, ZW. Investigation, XZ, SL, and DC. Project administration, SL, JZ, and ZW. Resources, DC, WZ, JZ, and ZW. Software, SL and YL. Validation, XZ, DC, SL, WZ, and YL. Visualization, SL and XW. Writing - original draft, XZ, DC, and SL. Writing - review and editing, XZ, JZ, and ZW. All authors contributed to the article and approved the submitted version.

## Funding

This study was supported by the National Natural Science Foundation (No. 81871986).

## Conflict of Interest

The authors declare that the research was conducted in the absence of any commercial or financial relationships that could be construed as a potential conflict of interest.

## Publisher’s Note

All claims expressed in this article are solely those of the authors and do not necessarily represent those of their affiliated organizations, or those of the publisher, the editors and the reviewers. Any product that may be evaluated in this article, or claim that may be made by its manufacturer, is not guaranteed or endorsed by the publisher.

## References

[1] Sung H Ferlay J Siegel RL Laversanne M Soerjomataram I Jemal A. Global Cancer Statistics 2020: GLOBOCAN Estimates of Incidence and Mortality Worldwide for 36 Cancers in 185 Countries. CA Cancer J Clin (2021) 71(3):209–49. doi: 10.3322/caac.21660 33538338

[2] GBD 2017 Oesophageal Cancer Collaborators. The Global, Regional, and National Burden of Oesophageal Cancer and its Attributable Risk Factors in 195 Countries and Territories, 1990-2017: A Systematic Analysis for the Global Burden of Disease Study 2017. Lancet Gastroenterol Hepatol (2020) 5(6):582–97. doi: 10.1016/S2468-1253(20)30007-8 PMC7232026 32246941

[3] Siegel RL Miller KD Fuchs HE Jemal A. Cancer Statistics, 2021. CA Cancer J Clin (2021) 71(1):7–33. doi: 10.3322/caac.21654 33433946

[4] Wang Q Peng L Han Y Li T Dai W Wang Y. Preoperative Serum Sodium Level as a Prognostic and Predictive Biomarker for Adjuvant Therapy in Esophageal Cancer. Front Oncol (2021) 10:555714. doi: 10.3389/fonc.2020.555714 33552948 PMC7858663

[5] Ilson DH van Hillegersberg R. Management of Patients With Adenocarcinoma or Squamous Cancer of the Esophagus. Gastroenterology (2018) 154(2):437–51. doi: 10.1053/j.gastro.2017.09.048 29037469

[6] Li SJ Wang ZQ Li YJ Fan J Zhang WB Che GW. Diabetes Mellitus and Risk of Anastomotic Leakage After Esophagectomy: A Systematic Review and Meta-Analysis. Dis Esophagus (2017) 30(6):1–12. doi: 10.1093/dote/dox006 28475743

[7] Yang J Liu X Cao S Dong X Rao S Cai K. Understanding Esophageal Cancer: The Challenges and Opportunities for the Next Decade. Front Oncol (2020) 10:1727. doi: 10.3389/fonc.2020.01727 33014854 PMC7511760

[8] Li S Wang H Yang Z Zhao L Lv W Du H. Naples Prognostic Score as a Novel Prognostic Prediction Tool in Video-Assisted Thoracoscopic Surgery for Early-Stage Lung Cancer: A Propensity Score Matching Study. Surg Endosc (2021) 35(7):3679–97. doi: 10.1007/s00464-020-07851-7 32748268

[9] Feng JF Zhao JM Chen S Chen QX. Naples Prognostic Score: A Novel Prognostic Score in Predicting Cancer-Specific Survival in Patients With Resected Esophageal Squamous Cell Carcinoma. Front Oncol (2021) 11:652537. doi: 10.3389/fonc.2021.652537 34123805 PMC8193841

[10] Li SJ Zhao L Wang HY Zhou HN Ju J Du H. Gustave Roussy Immune Score Based on a Three-Category Risk Assessment Scale Serves as a Novel and Effective Prognostic Indicator for Surgically Resectable Early-Stage Non-Small-Cell Lung Cancer: A Propensity Score Matching Retrospective Cohort Study. Int J Surg (2020) 84:25–40. doi: 10.1016/j.ijsu.2020.10.015 33086147

[11] Hao J Chen C Wan F Zhu Y Jin H Zhou J. Prognostic Value of Pre-Treatment Prognostic Nutritional Index in Esophageal Cancer: A Systematic Review and Meta-Analysis. Front Oncol (2020) 10:797. doi: 10.3389/fonc.2020.00797 32626652 PMC7311778

[12] Li SJ Lv WY Du H Li YJ Zhang WB Che GW. Albumin-To-Alkaline Phosphatase Ratio as a Novel Prognostic Indicator for Patients Undergoing Minimally Invasive Lung Cancer Surgery: Propensity Score Matching Analysis Using a Prospective Database. Int J Surg (2019) 69:32–42. doi: 10.1016/j.ijsu.2019.07.008 31319230

[13] Guner A Cho M Kim YM Cheong JH Hyung WJ Kim HI. Prognostic Value of Postoperative Neutrophil and Albumin: Reassessment One Month After Gastric Cancer Surgery. Front Oncol (2021) 11:633924. doi: 10.3389/fonc.2021.633924 33833991 PMC8023044

[14] Loftus TJ Brown MP Slish JH Rosenthal MD. Serum Levels of Prealbumin and Albumin for Preoperative Risk Stratification. Nutr Clin Pract (2019) 34(3):340–8. doi: 10.1002/ncp.10271 30908744

[15] An L Yin WT Sun DW. Albumin-To-Alkaline Phosphatase Ratio as a Promising Indicator of Prognosis in Human Cancers: Is it Possible? BMC Cancer (2021) 21(1):247. doi: 10.1186/s12885-021-07921-6 33685425 PMC7938577

[16] Chan AW Chan SL Mo FK Wong GL Wong VW Cheung YS. Albumin-To-Alkaline Phosphatase Ratio: A Novel Prognostic Index for Hepatocellular Carcinoma. Dis Markers (2015) 2015:564057. doi: 10.1155/2015/564057 25737613 PMC4337043

[17] Kim JS Keam B Heo DS Han DH Rhee CS Kim JH. The Prognostic Value of Albumin-To-Alkaline Phosphatase Ratio Before Radical Radiotherapy in Patients With Non-Metastatic Nasopharyngeal Carcinoma: A Propensity Score Matching Analysis. Cancer Res Treat (2019) 51(4):1313–23. doi: 10.4143/crt.2018.503 PMC6790835 30699498

[18] Tan P Xie N Ai J Xu H Xu H Liu L. The Prognostic Significance of Albumin-To-Alkaline Phosphatase Ratio in Upper Tract Urothelial Carcinoma. Sci Rep (2018) 8(1):12311. doi: 10.1038/s41598-018-29833-5 30120312 PMC6097991

[19] Xiong JP Long JY Xu WY Bian J Huang HC Bai Y. Albumin-To-Alkaline Phosphatase Ratio: A Novel Prognostic Index of Overall Survival in Cholangiocarcinoma Patients After Surgery. World J Gastrointest Oncol (2019) 11(1):39–47. doi: 10.4251/wjgo.v11.i1.39 30984349 PMC6451927

[20] Zhou S Jiang W Wang H Wei N Yu Q. Predictive Value of Pretreatment Albumin-to-Alkaline Phosphatase Ratio for Overall Survival for Patients With Advanced Non-Small Cell Lung Cancer. Cancer Med (2020) 9(17):6268–80. doi: 10.1002/cam4.3244 PMC7476831 32691996

[21] Li S Lu S Liu X Chen X. Association Between the Pretreatment Albumin-To-Alkaline Phosphatase Ratio and Clinical Outcomes in Patients With Bladder Cancer Treated With Radical Cystectomy: A Retrospective Cohort Study. Front Oncol (2021) 11:664392. doi: 10.3389/fonc.2021.664392 33959511 PMC8093628

[22] Haksoyler V Topkan E. Prognostic Utility of Prechemoradiotherapy Albumin-To-Alkaline Phosphatase Ratio in Unresectable Locally Advanced Pancreatic Carcinoma Patients. Gastroenterol Res Pract (2021) 2021:6647145. doi: 10.1155/2021/6647145 33927759 PMC8049825

[23] Acikgoz Y Bal O Dogan M. Albumin-To-Alkaline Phosphatase Ratio: Does It Predict Survival in Grade 1 and Grade 2 Neuroendocrine Tumors? Pancreas (2021) 50(1):111–7. doi: 10.1097/MPA.0000000000001720 33370032

[24] Tian G Li G Guan L Yang Y Li N. Pretreatment Albumin-to-Alkaline Phosphatase Ratio as a Prognostic Indicator in Solid Cancers: A Meta-Analysis With Trial Sequential Analysis. Int J Surg (2020) 81:66–73. doi: 10.1016/j.ijsu.2020.07.024 32745716

[25] Agha R Abdall-Razak A Crossley E Dowlut N Iosifidis C Mathew G. STROCSS 2019 Guideline: Strengthening the Reporting of Cohort Studies in Surgery. Int J Surg (2019) 72:156–65. doi: 10.1016/j.ijsu.2019.11.002 31704426

[26] Rice TW Ishwaran H Hofstetter WL Kelsen DP Apperson-Hansen C Blackstone EH. Recommendations for Pathologic Staging (pTNM) of Cancer of the Esophagus and Esophagogastric Junction for the 8th Edition AJCC/UICC Staging Manuals. Dis Esophagus (2016) 29(8):897–905. doi: 10.1111/dote.12533 27905172 PMC5591444

[27] Kataoka K Nakamura K Mizusawa J Kato K Eba J Katayama H. Surrogacy of Progression-Free Survival (PFS) for Overall Survival (OS) in Esophageal Cancer Trials With Preoperative Therapy: Literature-Based Meta-Analysis. Eur J Surg Oncol (2017) 43(10):1956–61. doi: 10.1016/j.ejso.2017.06.017 28747249

[28] Dindo D Demartines N Clavien PA. Classification of Surgical Complications: A New Proposal With Evaluation in a Cohort of 6336 Patients and Results of a Survey. Ann Surg (2004) 240(2):205–13. doi: 10.1097/01.sla.0000133083.54934.ae PMC1360123 15273542

[29] Fearon K Strasser F Anker SD Bosaeus I Bruera E Fainsinger RL. Definition and Classification of Cancer Cachexia: An International Consensus. Lancet Oncol (2011) 12(5):489–95. doi: 10.1016/S1470-2045(10)70218-7 21296615

[30] Anandavadivelan P Lagergren P. Cachexia in Patients With Oesophageal Cancer. Nat Rev Clin Oncol (2016) 13(3):185–98. doi: 10.1038/nrclinonc.2015.200 26573424

[31] Mantovani A Allavena P Sica A Balkwill F. Cancer-Related Inflammation. Nature (2008) 454(7203):436–44. doi: 10.1038/nature07205 18650914

[32] Namikawa T Ishida N Tsuda S Fujisawa K Munekage E Iwabu J. Prognostic Significance of Serum Alkaline Phosphatase and Lactate Dehydrogenase Levels in Patients With Unresectable Advanced Gastric Cancer. Gastric Cancer (2019) 22(4):684–91. doi: 10.1007/s10120-018-0897-8 30417313

